# A murine model of acute and prolonged abdominal sepsis, supported by intensive care, reveals time-dependent metabolic alterations in the heart

**DOI:** 10.1186/s40635-025-00715-1

**Published:** 2025-01-17

**Authors:** Bart Jacobs, Inge Derese, Sarah Derde, Sarah Vander Perre, Lies Pauwels, Greet Van den Berghe, Jan Gunst, Lies Langouche

**Affiliations:** Clinical Division and Laboratory of Intensive Care Medicine, Department of Cellular and Molecular Medicine, KU Leuven, Herestraat 49, O&N1 Box 503, 3000 Louvain, Belgium

**Keywords:** Sepsis, Sepsis-induced cardiomyopathy, Heart, Metabolism, Inflammation, Mitochondrial oxidation, Mitochondrial dysfunction

## Abstract

**Background:**

Sepsis-induced cardiomyopathy (SICM) often occurs in the acute phase of sepsis and is associated with increased mortality due to cardiac dysfunction. The pathogenesis remains poorly understood, and no specific treatments are available. Although SICM is considered reversible, emerging evidence suggests potential long-term sequelae. We hypothesized that metabolic and inflammatory cardiac changes, previously observed in acute sepsis as potential drivers of SICM, partially persist in prolonged sepsis.

**Methods:**

In 24-week-old C57BL/6J mice, sepsis was induced by cecal ligation and puncture, followed by intravenous fluid resuscitation, subcutaneous analgesics and antibiotics, and, in the prolonged phase, by parenteral nutrition. Mice were killed after 5 days of sepsis (prolonged sepsis, *n* = 15). For comparison, we included acutely septic mice killed at 30 h (acute sepsis, *n* = 15) and healthy controls animals (HC, *n* = 15). Cardiac tissue was collected for assessment of inflammatory and metabolic markers through gene expression, metabolomic analysis and histological assessment.

**Results:**

In prolonged sepsis, cardiac expression of IL-1β and IL-6 and macrophage infiltration remained upregulated (*p* ≤ 0.05). In contrast, tissue levels of Krebs cycle intermediates and adenosine phosphates were normal, whereas NADPH levels were low in prolonged sepsis (*p* ≤ 0.05). Gene expression of fatty acid transporters and of the glucose transporter *Slc2a1* was upregulated in prolonged sepsis (*p* ≤ 0.01). Lipid staining and glycogen content were elevated in prolonged sepsis together with increased gene expression of enzymes responsible for lipogenesis and glycogen synthesis (*p* ≤ 0.05). Intermediate glycolytic metabolites (hexose-phosphates, GADP, DHAP) were elevated (*p* ≤ 0.05), but gene expression of several enzymes for glycolysis and mitochondrial oxidation of pyruvate, fatty-acyl-CoA and ketone bodies to acetyl-CoA were suppressed in prolonged sepsis (*p* ≤ 0.05). Key metabolic transcription factors PPARα and PGC-1α were downregulated in acute, but upregulated in prolonged, sepsis (*p* ≤ 0.05 for both). Ketone body concentrations were normal but ketolytic enzymes remained suppressed (*p* ≤ 0.05). Amino acid metabolism showed mild, mixed changes.

**Conclusions:**

Our results suggest myocardial lipid and glycogen accumulation and suppressed mitochondrial oxidation, with a functionally intact Krebs cycle, in the prolonged phase of sepsis, together with ongoing myocardial inflammation. Whether these alterations have functional consequences and predispose to long-term sequelae of SICM needs further research.

**Supplementary Information:**

The online version contains supplementary material available at 10.1186/s40635-025-00715-1.

## Background

Patients suffering from sepsis can develop specific sepsis-induced cardiomyopathy (SICM). Its reported prevalence in septic patients ranges from 10 to 70% [1] depending on the diagnostic criteria being used, with a recent meta-analysis reporting a pooled prevalence of 20% [2]. The presence of SICM is clinically relevant, as mortality is associated with systolic [3] and diastolic [4] cardiac dysfunction. Although there is no consensus on clear diagnostic criteria, SICM is defined as acute cardiac dysfunction associated with sepsis, unrelated to myocardial ischemia [5]. In fact, coronary perfusion often increases in patients with septic shock [6]. Typical characteristics include left ventricular dilatation, impaired ventricular contractility, and/or dysfunction of both right and left ventricles with fluid unresponsiveness [1, 5, 7]. However, distinguishing intrinsic cardiac dysfunction from abnormalities in vascular and autonomic status remains challenging [8].

Despite its clinical association with poor outcomes [2, 5, 9], the pathogenesis of SICM remains poorly understood, and no specific treatments are available. There are relatively few arguments for significant structural damage, and cell death does not seem to be a major driver of SICM [10–12]. Instead, infiltration of immune cells into the myocardium, fluid accumulation, and mitochondrial damage seem to be the main pathological findings in SICM [10]. This suggests a key pathophysiologic role for inflammation and cellular bioenergetic failure. The inflammatory response to acute sepsis is characterized by high levels of circulating cytokines, presence of pathogen- and damage-associated molecular patterns (PAMPs and DAMPs), excessive nitric oxide formation and elevated reactive oxygen species [7, 13], which likely exacerbate cardiac dysfunction. Mitochondrial dysfunction, documented in experimental models of SICM, includes mitochondrial uncoupling, reduced oxygen consumption, and ATP depletion during the very acute (first 6 h) to acute (first 48 h) phases of sepsis [8]. These mitochondrial alterations have been interpreted as a cause of ‘energetic failure’, but also as an adaptation to reduce metabolic demands [7]. Mitochondrial dysfunction may partially explain alterations in cardiac metabolism during acute sepsis [6, 12], although a shift in substrate availability and the hormonal stress response might also play a role [5, 7, 14]. During health, 60–90% of ATP in the heart is generated by fatty acid oxidation, and 40–10% by glucose–pyruvate oxidation [15]. In contrast, patients with septic shock appear to accumulate myocardial lipids [12] and shift to glycolysis [6]. Experimental models of acute sepsis described suppressed myocardial fatty acid oxidation, lipid accumulation, as well as increased glucose uptake, glycogen deposition, and pyruvate dehydrogenase (PDH) inactivation, indicative of suppressed glucose oxidation [16–18].

However, it is unclear whether the inflammatory changes, along with mitochondrial and metabolic alterations, are temporary or persist in more prolonged sepsis. The clinical features of septic cardiomyopathy appear to be transient and are particularly associated with the acute hyperinflammatory phase of sepsis [19–21]. Some experts have incorporated this apparent transient nature into the definition of septic cardiomyopathy [22]. Nevertheless, the reversibility of SICM has not been conclusively demonstrated in large observational studies [8, 23]. Moreover, the metabolic response to sepsis causes a disturbed total body metabolism with high levels of catabolic hormones and an anabolic resistance also in the more prolonged phase of sepsis [24, 25]. Furthermore, emerging evidence suggests that SICM may have long-term clinical consequences. Right ventricular dysfunction during sepsis has been associated with both short- and long-term (>30 days) mortality in a meta-analysis of observational data [26]. Additionally, among ICU survivors of sepsis or septic shock, a U-shaped association was found between left ventricular global strain—a marker of systolic function—at ICU admission and cardiovascular events within 24 months after discharge [27].

Given the possibility of long-term sequelae of SICM, we hypothesized that the shift in myocardial metabolism and the inflammatory changes observed during acute sepsis at least partially persist in prolonged sepsis, alongside other metabolic changes specific to prolonged sepsis. Therefore, we documented sepsis-induced metabolic and inflammatory changes in the myocardium over time with use of an experimental model. We used a well-validated, clinically relevant mouse model of sepsis induced by cecal ligation and puncture (CLP) supported by intravenous fluid resuscitation, analgesics and antibiotics, and, in the prolonged phase, by parenteral nutrition. Metabolic alterations were studied via gene expression of key markers of fatty acid, glucose, protein and ketone body metabolism and quantification of key metabolites via histology and liquid chromatography–mass spectrometry.

## Methods

### Experiments in the mouse model of acute and prolonged sepsis

To study the impact of duration of sepsis (mouse study 1), 24-week-old male C57BL/6J mice (mature adult) (Janvier Labs, Le Genest-Saint-Ilse, France) were randomly allocated to ‘healthy control’ (day 0 mice) or ‘sepsis’. Septic mice were killed after 30 h (day 1, acute sepsis), or 5 days (day 5, prolonged sepsis). We used only male mice to avoid the cyclic influence of estrogens. The Animal Ethics Committee of KU Leuven approved the protocol (P134-2013). The study adhered to the European Union’s Directive 2010/63/EU concerning the welfare of laboratory animals and complied with the ARRIVE guidelines [28]. Mice randomized to sepsis were anesthetized with xylazine/ketamine and the left internal jugular vein was cannulated, followed by a median laparotomy and CLP to induce polymicrobial abdominal sepsis (described in detail in [29]). In the first 24 h after surgery, septic mice received continuous intravenous fluid resuscitation (4:1 mixture of crystalloids/colloids) at 0.3 ml/h. Hereafter, parenteral nutrition (Oliclinomel N7E, Baxter, Braine-l’Alleud, Belgium) was administrated at 0.2 ml/h (up to 45% of the daily caloric intake in health, to mimic the illness-induced lack of feeding in human sepsis patients) until the end of the study period. All septic mice were treated with broad-spectrum antibiotics (imipenem/cilastatin, Aurobindo Pharma, Hyderabad, India) and opioid-analgesics (buprenorphine (Vetergesic), Patheon UK Ltd, Covingham, United Kingdom) via subcutaneous injection twice daily. If the central venous catheter was accidently dislocated, mice were excluded. Healthy control mice did not receive any intervention and received ad libitum feeding and free access to water. All animal cages were kept in an animal cabinet under controlled temperature (27 °C) and 12 h light and dark cycles.

In a second experiment, aimed to perform metabolomics on cardiac tissue of prolonged septic mice, the experimental setup was repeated (ethical approval P135-2020), but now only mice surviving until day 5 and a healthy control group were included. Metabolomic concentrations were expressed relative to the average values observed in the healthy controls, providing a standardized reference for comparison across groups.

Both studies were continued until samples of at least 15 surviving animals per study group were obtained. Mortality was 6.25% for day 1 animals and 20% for day 5 animals in study 1 and 12% in study 2. After the planned study period of 1 or 5 days, mice were killed via cardiac puncture and whole blood and hearts were collected. The heart was immediately weighed, rinsed in saline solution and the apical zone (±25 mg) was snap-frozen and stored at −80 °C for later gene expression analysis or metabolomic analysis. The mid-ventricular zone was stored in Tissue-Tek O.C.T. Compound (Sakura, Torrance, CA, USA) for cryosectioning and OilRedO staining, and the basal zone was fixated in paraformaldehyde and later embedded in paraffin for histological sectioning.

### Myocardial tissue analyses

From stored frozen cardiac tissue, genomic DNA was removed by DNAse treatment (Macherey-Nagel Nucleospin RNA kit [Macherey–Nagel, Düren, Germany]). RNA was reverse-transcribed with the use of random hexamers (Invitrogen, Waltham, MA, USA). Next, cDNA was quantified in real time with the use of commercial TaqMan assays (Applied Biosystems Waltham, MA, USA). Relative gene expression was determined with the 2−ΔΔCt method with 18S ribosomal RNA (Rn18s) as housekeeping gene (Applied Biosystems) and are presented as fold difference of the mean of the healthy controls. An overview of the gene expression assays used is provided in Supplementary Table 1. The presence of macrophages was assessed with immunohistochemistry. In short, paraffin cross-sections of cardiac tissue were incubated overnight at 4°C with an anti-CD68 primary antibody (Abcam, Cambridge, UK; RRID: AB_10975465) and subsequently with a secondary HRP-linked antibody (Dako, Glostrup, Denmark), followed by visualization with diaminobenzidine. Images were captured with a Leica DM3000 bright-field microscope (Leica Camera, Wetzlar, Germany) and digital camera with LAS V4.10 software (Heerbrugg, Switzerland). Positive staining was semi-quantitatively assessed by 2 independent investigators on a scale from 0 (no staining) to 2 (intensive positive staining). Cryosections were used for visualization of neutral lipid accumulation with OilRedO-staining (ORO, Sigma-Aldrich, St Louis, USA) and semi-quantitatively analyzed for the relative amount of redness (score 0 [lipid accumulation not present], score 1 [lipid accumulation somewhat present], score 2 [lipid accumulation clearly present]) by 2 independent investigators.

For the metabolomics of the second mouse experiment, approximately 10 mg of cardiac tissue was homogenized in 800 µl extraction buffer (twice at 6500 rpm for 30 s). The tubes were kept overnight at −80 °C. On the next day, the samples were centrifuged for 10 min at 4 °C and maximum rpm, and the supernatant was transferred to MS vials. Ten µl of sample was loaded into a Dionex UltiMate 3000 LC System (Thermo Scientific Bremen, Germany) equipped with a C-18 column (Acquity UPLC-HSS T3 1. 8 µm; 2.1 × 150 mm, Waters) coupled to a Q Exactive Orbitrap mass spectrometer (Thermo Scientific) operating in negative ion mode. A step gradient was carried out using solvent A (10 mM TBA and 15 mM acetic acid) and solvent B (100% methanol). Data collection was performed using the Xcalibur software (Thermo Scientific). The data analyses were performed by integrating the peak areas (El-Maven—Polly—Elucidata).

### Statistical analyses

Data were compared with Kruskal–Wallis tests, and, where appropriate, additional post hoc Wilcoxon rank-sum tests were performed to compare the different timepoints. Two-sided *P*-values below 0.05 were considered statistically significant. No corrections for multiple comparisons were performed. All analyses were performed with JMP Pro 17.0.0 (SAS Institute Inc., Cary, NC, USA). Data are presented as median (interquartile range, IQR) in box–whisker plots, with the horizontal line within the box representing the median, the box the 25th and 75th quantiles, and the whiskers indicating the furthest point within 1.5 times this IQR from the end of the box.

## Results

### Markers of inflammation and energy homeostasis

Gene expression of cytokines IL-1β (Il1b) and IL-6 (Il6), but not Tnf (Tnf) was upregulated in acute and prolonged sepsis (Fig. 1.1). Immunostaining for macrophage marker CD68 showed increased positive staining in acute and prolonged sepsis (Fig. 1.2).

**Fig. 1:**
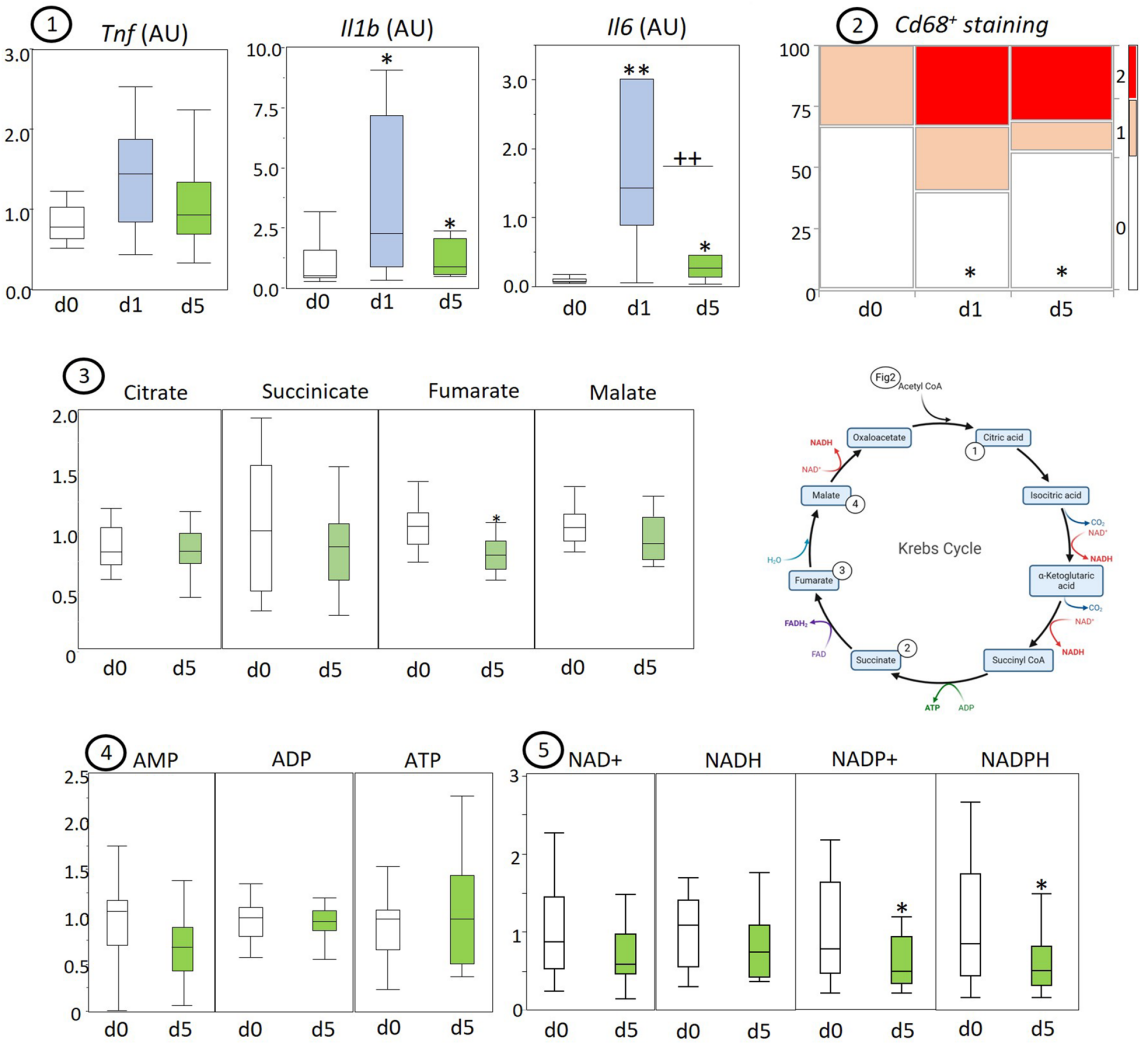
Myocardial markers of inflammation and energy homeostasis in healthy control (d0), acute septic (d1), and prolonged septic mice (d5). Data on enzyme expressions and concentrations of metabolic intermediates are presented in box and whisker plots. CD68 immunostaining is shown through an intensity heatmap. The schematic diagram created with Biorender illustrates the metabolic pathways involved where green numbers indicate increased markers and red numbers decreased markers in prolonged sepsis. (1) Gene expression of tumor necrosis factor-alpha (TNF-α), interleukin-1 beta (IL-1β), interleukin-6 (IL-6); (2) CD68 immunostaining intensity heatmap, analyzed semi-quantitatively for macrophage presence with 0—no macrophage staining, 1—moderate presence, 2—high presence; LCMS quantified (3) Krebs cycle intermediates: citrate, succinate, malate, and fumarate, (4) adenosine mono-phosphate (AMP), adenosine di-phosphate (ADP), and adenosine tri-phosphate (ATP) and (5) electron carriers nicotinamide adenine dinucleotide NAD+/NADH and nicotinamide adenine dinucleotide phosphate NADP+/NADPH. Metabolite levels are expressed relative to the average of healthy controls. *P ≤ 0.05 compared with healthy control animals. *IQR* interquartile range, *a.u.* arbitrary unit

Quantification during prolonged sepsis of myocardial Krebs cycle intermediates (Fig. 1.3) showed normal levels for citrate, succinate and malate, and a slight but significant reduction in tissue concentration for fumarate. Myocardial adenosine mono-, di- and tri-phosphate concentrations were normal in prolonged sepsis (Fig. 1.4). Also, the concentration of electron carrier pair NAD+/NADH was not affected, whereas both NADP+ and NADPH were decreased in prolonged sepsis (Fig. 1.5).

### Markers of fatty acid metabolism

Gene expression of fatty acid uptake transporters Cd36 (Cd36) and Fatp1 (Slc27a1) increased with sepsis and stayed elevated during prolonged sepsis (Fig. 2.1, 2.2). Intracellular fatty acids (myristic acid, palmitic acid and stearic acid) and triglyceride concentrations (Fig. 2.3) were not altered at day 5 of sepsis, whereas OilRedO staining, indicative of neutral lipid storage, tended to be increased in acute and prolonged sepsis (*p* = 0.06; Fig. 2.4). Remarkably, gene expression of the fatty-acyl-CoA inactivating enzyme acyl-CoA thiosterase 1 (Acot1) was significantly increased in acute sepsis (d1), and remained elevated during prolonged sepsis, although to a lesser extent (Fig. 2.5). Expression of the key nuclear transcription factor regulating lipid metabolism, PPARα (PPARa; Fig. 2.6) and its co-activator PGC1α (Ppargc1a; Fig. 2.7) were downregulated in acute sepsis, but increased above healthy levels in prolonged sepsis. Gene expression of CPT-1b (Cpt1b), responsible for the mitochondrial uptake of fatty-acyl-CoA was not affected (Fig. 2.8), whereas expression of the dehydrogenase enzymes (Acadm, Acadl), required for the conversion to acetyl-CoA, were downregulated in prolonged sepsis (Fig. 2.9). LC–MS quantification of acetyl-CoA and CoA with metabolomics showed unaltered concentrations in the myocardium of prolonged septic mice (Fig. 2.10).

**Fig. 2:**
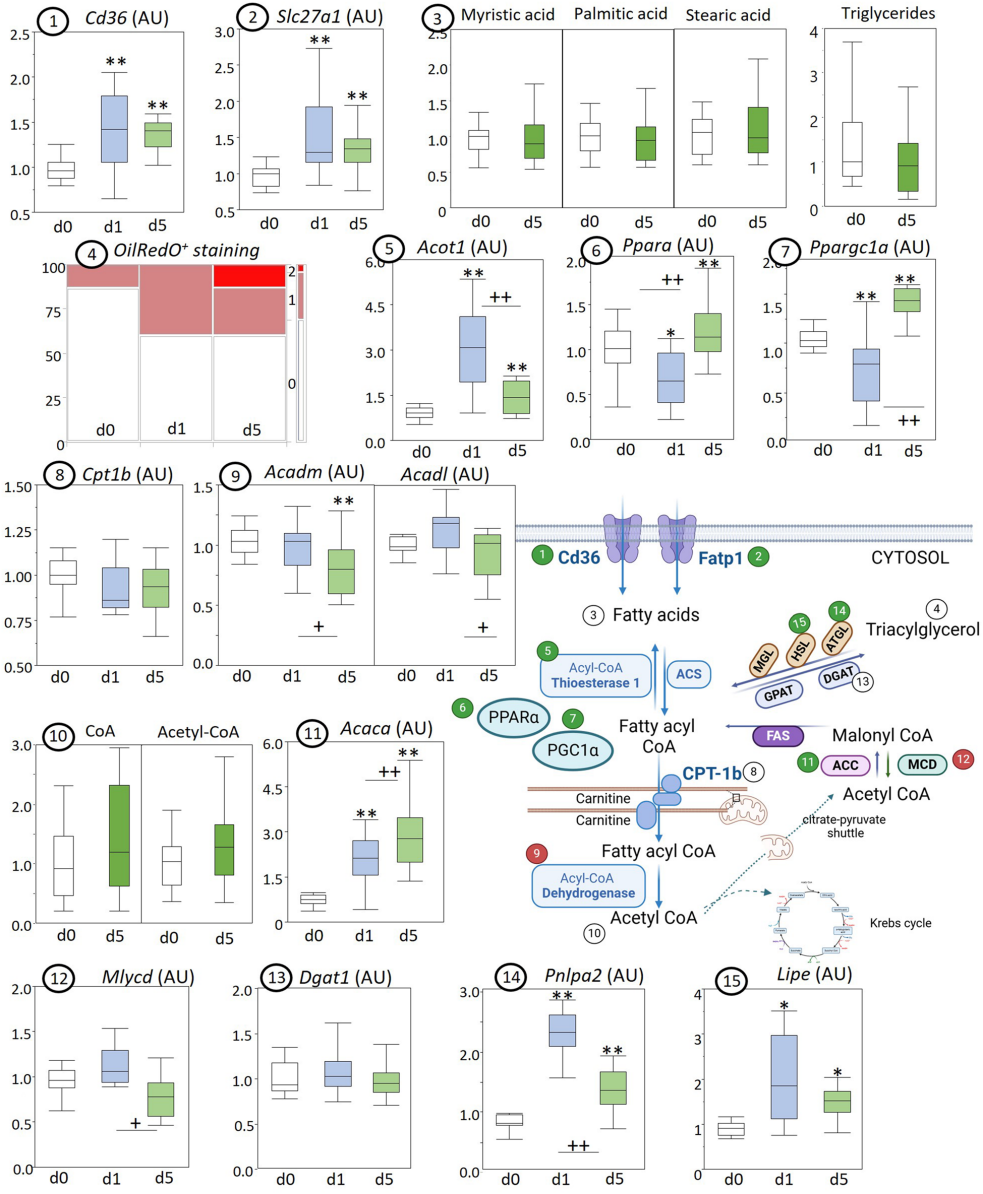
Myocardial markers of fatty acid metabolism in from healthy control (d0), acute septic (d1), and prolonged septic mice (d5). Enzyme expressions and metabolic intermediates are presented in box and whisker plots. OilRedO staining is shown through an intensity heatmap. The schematic diagram created with Biorender illustrates the pathways involved where green numbers indicate increased markers and red numbers decreased markers in prolonged sepsis. (1) Gene expression of Fatty acid transporters CD36 and (2) Slc27a1; (3) LCMS quantified myristic acid, palmitic acid, stearic acid, and triglycerides; (4) OilRedO staining intensity heatmap, semi-quantitatively analyzed for relative redness with 0—no or low lipid accumulation, 1—moderate lipid accumulation, 2—clear lipid accumulation; gene expression of (5) acyl-CoA thioesterase 1 (Acot1), (6) peroxisome proliferator-activated receptor alpha (Ppara) and (7) its co-activator peroxisome proliferator-activated receptor gamma co-activator 1-alpha (Ppargc1a); (8) carnitine palmitoyltransferase 1 (CPT1b), (9) dehydrogenase enzymes acyl-CoA dehydrogenase medium chain (Acadm) and acyl-CoA dehydrogenase long chain (Acadl); (10) LCMS quantified coenzyme A (CoA) and acetyl-coenzyme A (acetyl-CoA); (11) gene expression of acetyl-CoA carboxylase alpha (Acaca), (12) malonyl-CoA decarboxylase (Mlcyd), (13) diacylglycerol O-acyltransferase 1 (Dgat1), (14) adipose triglyceride lipase (Pnlpa2) and (15) hormone-sensitive lipase (Lipe). Metabolite levels are expressed relative to the average of healthy controls. *P ≤ 0.05/**P ≤ 0.01 compared with healthy control animals, + +*P* ≤ 0.01 compared between d1 and d5; *IQR* interquartile range, *a.u.* arbitrary unit

Expression of the flux-controlling enzyme of fatty acid synthesis, acetyl-CoA-carboxylase A (Acaca), was upregulated (Fig. 2.11), whereas gene expression of malonyl-CoA-decarboxylase (Mlycd) was downregulated (Fig. 2.12) in prolonged sepsis. Expression of lipogenic enzyme DGAT1 (Dgat1) was not altered (Fig. 2.13), but lipolytic enzymes ATGL (Pnlpa2; Fig. 2.14) and HSL (Lipe; Fig. 2.15) displayed elevated expression both in acute and prolonged sepsis.

### Markers of glucose metabolism

Gene expression of glucose transporter GLUT1 (Slc2a1), but not GLUT4 (Slc2a4), was upregulated in acute and prolonged sepsis (Fig. 3.1). The concentration of hexose was decreased in prolonged sepsis, but hexose-phosphates as a group (glucose-6-phosphate, glucose-1-phosphate, fructose-6-phosphate) and glycogen were increased (Fig. 3.2–3.4). Expression of glycogen synthase 1 (Gys1) was upregulated in acute and prolonged sepsis (Fig. 3.5), whereas expression of regulator glycogen synthase kinase (Gsk3b; Fig. 3.6) or glycogen phosphorylase (Pygb; Fig. 3.7) were not affected. Gene expression of phosphofructokinase 1 was downregulated during acute and prolonged sepsis (Pfkm; Fig. 3.8), but hexose-biphosphate (Hexose-2P; Fig. 3.9) was normal and PFKFB2 (Pfkfbp2), the heart-specific enzyme responsible for the conversion to fructose-2,6-biphosphate, showed decreased expression in acute and prolonged sepsis (Fig. 3.10). In contrast, PFKB3 (Pfkfbp3) expression was increased (Fig. 3.11). Intermediate metabolites glyceraldehyde-3-phophate (Fig. 3.12) and dihydroxyacetone phosphate (Fig. 3.13) were elevated in the myocardium in prolonged sepsis, whereas pyruvate (Fig. 3.14) and lactate (Fig. 3.15) were not. Gene expression of lactate dehydrogenase (Ldhb; Fig. 3.16) and of the mitochondrial pyruvate carriers (Mpc1 and Mpc2; Fig. 3.17) was also not affected by sepsis. Gene expressions of enzymes of the pyruvate dehydrogenase complex (Pdha1, Pdhb) were unaffected or decreased throughout sepsis (Fig. 3.18), whereas pyruvate dehydrogenase kinase 3 (Pdk3), but not 1 (Pdk1), was upregulated (Fig. 3.19).

**Fig. 3:**
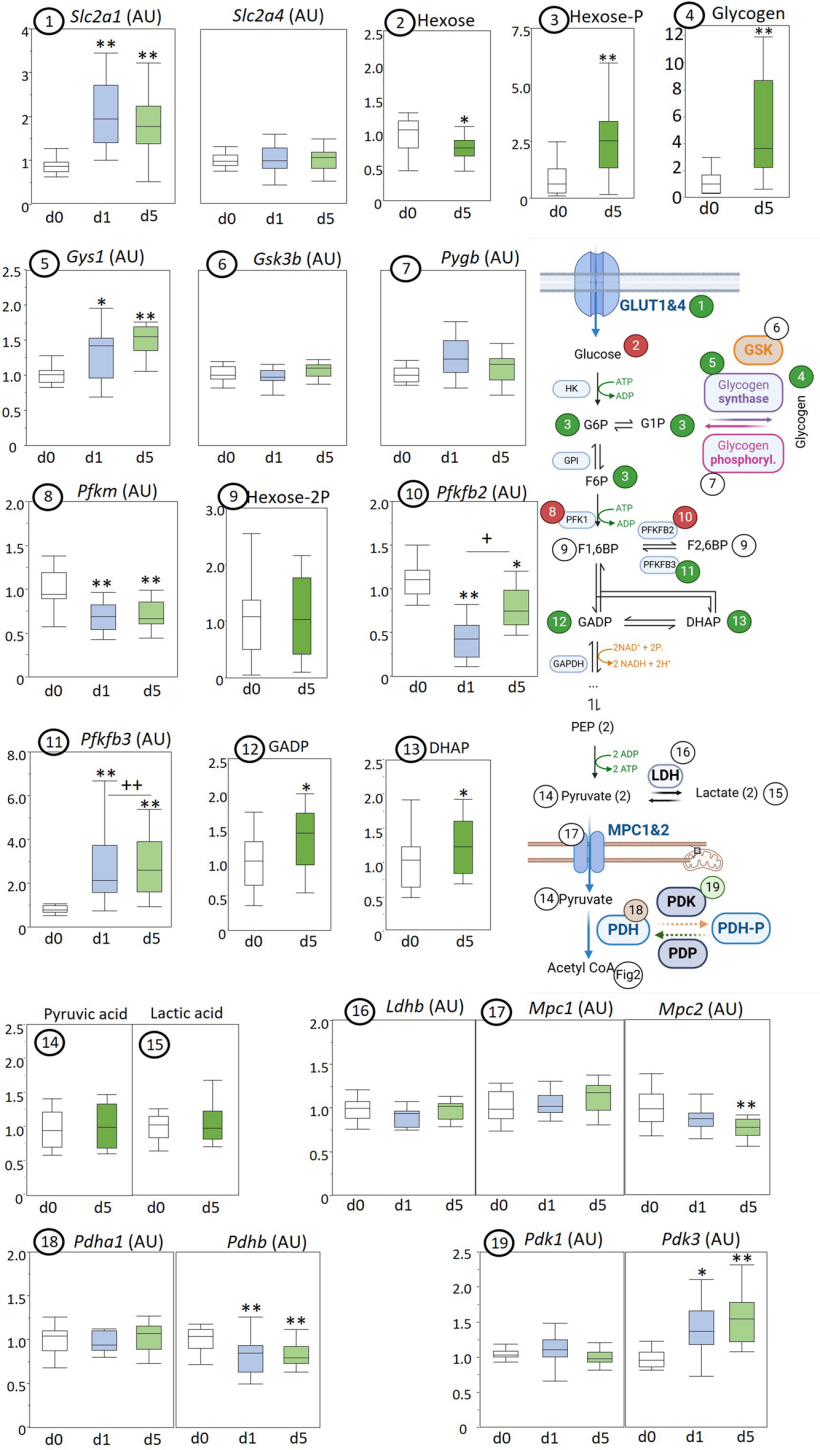
Myocardial markers of glucose metabolism in healthy control (d0), acute septic (d1), and prolonged septic mice (d5). Enzyme expressions and metabolic intermediates are presented in box and whisker plots. The schematic diagram created with Biorender illustrates the pathways involved where green numbers indicate increased markers and red numbers decreased markers in prolonged sepsis. Gene expression of (1) glucose transporter 1 (Slc2a1) and glucose transporter 4 (Slc2a4); (2) LCMS quantified hexose, (3) hexose-6-phosphate, and (4) glycogen; gene expression of (5) glycogen synthase 1 (Gys1), (6) glycogen synthase kinase 3 beta (Gsk3b), (7) glycogen branching enzyme (Pygb), (8) phosphofructokinase, muscle (Pfkm); (9) LCMS quantified hexose-bisphosphate; gene expression of (10) phosphofructokinase-2 (Pfkfbp2) and (11) phosphofructokinase-3 (Pfkfbp3); LCMS quantified (12) glyceraldehyde-3-phosphate (GADP) and (13) dihydroxyacetone phosphate (DHAP), (14) pyruvate and (15) lactate; gene expression of (16) lactate dehydrogenase B (Ldhb), (17) mitochondrial pyruvate carriers 1 and 2 (Mpc1 and Mpc2), (18) pyruvate dehydrogenase E1 component subunit alpha 1 (Pdha1) and pyruvate dehydrogenase E1 component subunit beta (Pdhb), (19) pyruvate dehydrogenase kinase 1 (Pdk1), and pyruvate dehydrogenase kinase 3 (Pdk3). Metabolite levels are expressed relative to the average of healthy controls. *P ≤ 0.05/**P ≤ 0.01 compared with healthy control animals, +*P* ≤ 0.05 /++*P* ≤ 0.01 compared between d1 and d5; *IQR* interquartile range, *a.u.* arbitrary unit

### Markers of ketone metabolism

The myocardial concentrations of ketone body 3-hydroxybutyrate (Fig. 4.1) and acetoacetate (Fig. 4.3) were unaltered in prolonged sepsis, but gene expression of the converting enzyme 3-hydroxybutyrate dehydrogenase (Bdh1; Fig. 4.2) was increased in acute and prolonged sepsis. Gene expression of ketolytic enzymes SCOT (Oxct1; Fig. 4.4) and ACAT (Acat1; Fig. 4.5) was downregulated in acute and prolonged sepsis, whereas expression of ketogenic enzyme HMGCS2 (hmgcs2) was upregulated in acute, but not in prolonged sepsis (Fig. 4.6).

**Fig. 4:**
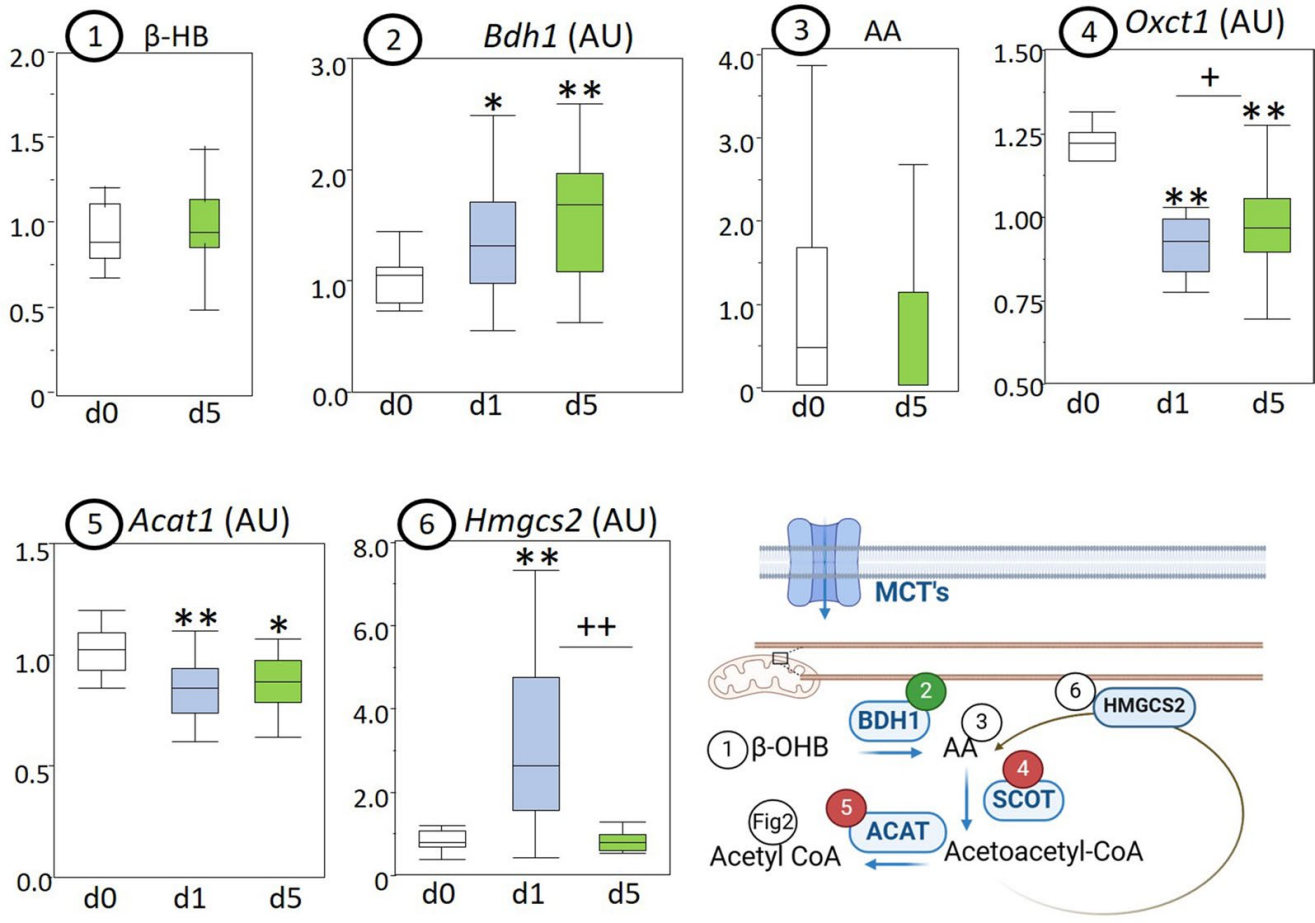
Myocardial markers of myocardial ketone metabolism in healthy control (d0), acute septic (d1), and prolonged septic mice (d5). Data on enzyme expressions and concentrations of ketone bodies are presented in box and whisker plots. The schematic diagram created with Biorender illustrates the pathways involved where green numbers indicate increased markers and red numbers decreased markers in prolonged sepsis. (1) LCMS quantified ketone body 3-hydroxybutyrate (β-OHB); (2) gene expression of 3-hydroxybutyrate dehydrogenase (Bdh1); (3) LCMS quantified ketone body acetoacetate (AA); gene expression of (4) succinyl-CoA:3-ketoacid coenzyme A transferase 1 (Oxct1), (5) acetoacetyl-CoA thiolase (Acat1) and (6) 3-hydroxy-3-methylglutaryl-CoA synthase 1 (Hmgcs2). Metabolite levels are expressed relative to the average of healthy controls. *P ≤ 0.05/**P ≤ 0.01 compared with healthy control animals, +*P* ≤ 0.05/++*P* ≤ 0.01 compared between d1 and d5; *IQR* interquartile range, *a.u.* arbitrary unit

### Markers of amino acid metabolism

The myocardial concentration of amino acids showed a mixed image in prolonged sepsis with unaltered levels for alanine, glycine, proline, serine, asparagine, cysteine, and histidine (Fig. 5.1). The concentrations of aromatic amino acids tryptophan and phenylalanine were increased and of tyrosine decreased. Glutamine concentration was also increased, whereas glutamic acid and aspartic acid were decreased. Also increased levels for methionine, threonine, lysine and arginine were observed in the myocardium in prolonged sepsis (Fig. 5.1). Branched-chained amino acids isoleucine and valine (Fig. 5.2) and gene expression of specific dehydrogenase subunits (Bckdha and Bckdb; Fig. 5.3) were unaltered, whereas gene expression of its regulator, branched chain ketoacid dehydrogenase kinase (Bckdk; Fig. 5.4), was decreased in acute and prolonged sepsis.

**Fig. 5:**
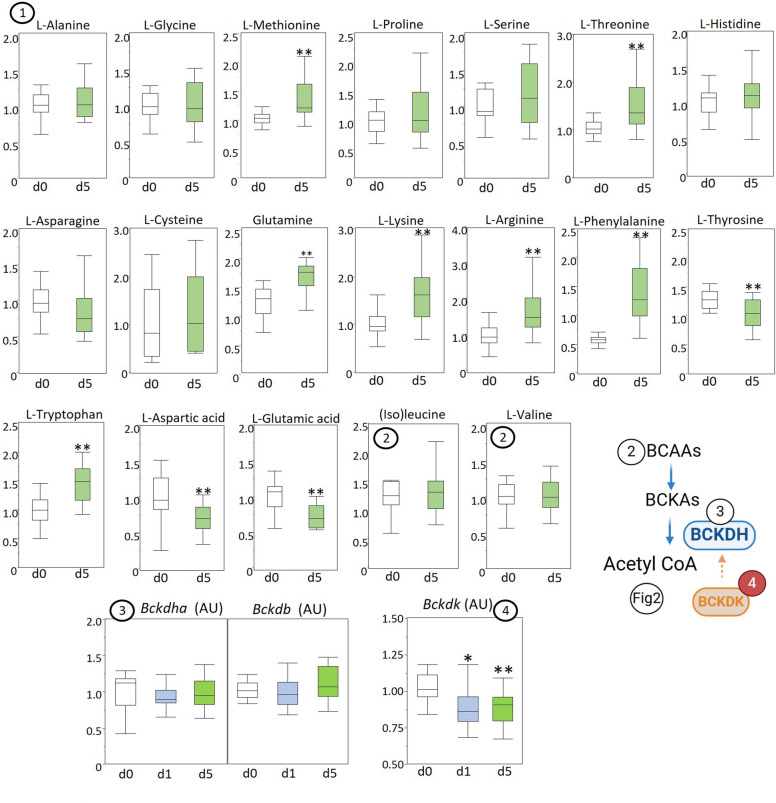
Myocardial markers of amino acid metabolism in healthy control (d0), acute septic (d1), and prolonged septic mice (d5). Amino acid concentrations and gene expressions are presented in box and whisker plots. The schematic diagram created with Biorender illustrates the amino acid metabolism pathways involving the discussed enzymes and substrates where green numbers indicate increased markers and red numbers decreased markers in prolonged sepsis. (1) LCMS quantified alanine, glycine, proline, serine, asparagine, cysteine, histidine, tryptophan, phenylalanine, tyrosine, glutamine, glutamic acid, aspartic acid, methionine, threonine, lysine, and arginine; (2) LCMS quantified branched-chain amino acids isoleucine and valine; gene expression of (3) branched-chain keto acid dehydrogenase enzyme subunits (Bckdha, Bckdb), and (4) branched chain ketoacid dehydrogenase kinase (Bckdk). Metabolite levels are expressed relative to the average of healthy controls. *P ≤ 0.05/**P ≤ 0.01 compared with healthy control animals, +*P* ≤ 0.05/++*P* ≤ 0.01 compared between d1 and d5; *IQR* interquartile range, *a.u.* arbitrary unit

## Discussion

This study performed in a murine model of sepsis revealed that the acute myocardial metabolic and inflammatory changes largely persisted in the prolonged phase. We primarily observed signs of reduced mitochondrial function, with markers suggesting a potential decrease in the oxidation of glucose, fatty acids, and ketone bodies to acetyl-CoA, and accumulation of energy substrates in the cytosol. Notably, the expression of PPARα and PGC-1α was suppressed during the acute phase of sepsis, but increased to supranormal levels in prolonged sepsis. This pattern suggests a transient suppression of fatty acid oxidation, followed by a compensatory response that may aim to restore metabolic function.

We observed a myocardial upregulation in expression of inflammatory cytokines IL-1β and IL-6 along with increased macrophage staining, which was more pronounced in acute than in prolonged sepsis. This aligns with human autopsy studies showing macrophage infiltration in sepsis [12]. Our findings are also consistent with mice studies of acute CLP-induced sepsis (24 h) and of those reporting changes 28 days post-sepsis [30, 31]. However, in our model, Tnf expression was not upregulated. This differs from some previous reports in animals and humans [7], although Zeng et al. found that Tnf only peaked in the hyperacute phase of CLP-induced sepsis, followed by normalization at 24 h [32]. Inflammatory cytokines likely play a crucial role in the pathogenesis of SICM, affecting cardiomyocyte contractility, as illustrated in vitro and in isolated rat hearts [7, 33–35] and in mice studies intervening with the NLRP3-IL-1β or IL-6 pathway [36–38]. Also in children suffering from meningococcal sepsis, serum IL-6 levels were predictive of the degree of myocardial dysfunction [39]. Together with our observations showing sustained myocardial inflammation in prolonged sepsis, the data indicate that inflammation could indeed be a potential driver of long-term sequelae of SICM. On the other hand, an increased macrophage infiltration has previously been suggested to reflect a defensive mechanism to help remove dysfunctional mitochondria from cardiomyocytes [40] and IL-6 knockout mice exhibited arrhythmia and increased oxidative stress upon LPS injection [41].

Both lipid and glycogen appeared to accumulate in the myocardial cytosol over time, as indicated by the OilRedO staining, glycogen concentrations, and increased gene expression of enzymes responsible for lipogenesis and glycogen synthesis. As triglyceride and free fatty acid myocardial concentrations appeared normal in prolonged sepsis, this suggests that the observed OilRedO staining might be caused by accumulation of cholesterol esters, di- or mono-glycerides. Potentially, the simultaneous increase in expression of lipolytic enzymes in early and prolonged sepsis might be a compensatory reaction to an increased lipid flux. In literature, glycogen accumulation was also documented in a CLP mouse model 48 h after sepsis induction [16], and lipid accumulation has been documented in prolonged human sepsis [12] and in mice, 6–8 h after LPS injection [17, 42].

An increased glycogen or lipid accumulation in the cytosol might be explained by decreased downstream mitochondrial function. Indeed, the gene expression markers of mitochondrial oxidation of pyruvate, fatty-acyl-CoA and ketone bodies to acetyl-CoA appeared suppressed in acute and prolonged sepsis in our model. Similar changes in gene expression, indicating suppressed mitochondrial oxidation, have been observed previously 6–8 h after LPS injection [42–44] and 24 h after CLP-induced sepsis in mice [18]. However, acetyl-CoA and most downstream TCA intermediates remained largely unaffected in prolonged sepsis. Of note, the enzyme responsible for converting acetyl-CoA to malonyl-CoA as the first step of lipogenesis was upregulated in acute and prolonged sepsis. Potentially also an increase in amino acid oxidation plays a role in the sustained acetyl-CoA concentration, as the gene expression of the oxidation-inhibiting enzyme BCKDK was suppressed in acute and prolonged sepsis. Substrate accumulation in the cytosol in the context of suppressed mitochondrial oxidation can be interpreted as relative overfeeding. Although mice received parenteral nutrition amounting to only 45% of their normal daily caloric intake under healthy conditions, the sepsis-induced catabolic state could have led to substrate overabundance. Whether an even more restrictive feeding strategy could improve myocardial mitochondrial function, as demonstrated in liver tissue of critically ill rabbits, requires further investigation [45].

As expected, given the unaltered concentrations of acetyl-CoA and almost all TCA intermediates, the concentration of ATP, ADP and AMP and NAD(H) remained unchanged in prolonged sepsis, although we did observe lower NADP(H) concentrations. In contrast to NAD(H), which is involved in ATP generation, NADP(H) is required for reductive de novo biosynthesis and antioxidant defense, but requires TCA cycle intermediates to be generated. Together these findings suggest a functionally intact but overall suppressed TCA cycle in prolonged sepsis. This is comparable with previously observed normal levels of TCA cycle intermediates and high-energy substrates measured 36–48 h after sepsis induction in CLP models [46, 47]. In contrast, in the very acute phase of CLP-induced sepsis (12–14 h) or after LPS injection, ATP depletion has been reported [48–50]. The downregulation of genes responsible for substrate oxidation, with a seemingly functionally intact TCA cycle and ATP production in our experiment, might be explained by the phenomenon of myocardial hibernation. Although primarily described in cardiac ischemia and hypoxia, this mechanism, characterized by dysfunctional but viable myocardium is also hypothesized to play a role in sepsis [8, 16]. Suppressed mitochondrial oxidation, likely driven by excessive inflammation, oxidative and metabolic stress might be responsible for this process, and could lead to a downregulation of substrate metabolism, which in turn creates a negative feedback loop to the mitochondrion. This metabolic bioenergetic shutdown could be protective during prolonged inflammatory hits by inhibiting cell death pathways [51].

The observed downregulated expression of PPARα and its co-activator PGC-1α in the acute phase of sepsis further supports an acute suppression of mitochondrial oxidation. These nuclear transcription factors are known to stimulate fatty acid oxidation and ketogenesis, while also reducing glucose sensitivity. In several studies in mice, the expression of PPARα and PGC-1α was also suppressed 6–16 h after LPS injection [17, 42, 43, 52]. Intervention studies in rodents point to a possible causal link between altered PPARα function and myocardial dysfunction in acute sepsis [17, 42, 43, 52, 53]. Moreover, a reinforcement mechanism, in which PGC-1α suppression by increased lipid accumulation leads to further suppression of mitochondrial oxidation and cytosolic lipid accumulation, could exacerbate myocardial dysfunction [15]. However, in prolonged sepsis, we observed an upregulation of these regulatory transcription factors, whereas mitochondrial oxidation to acetyl-CoA seemed to be suppressed. Potentially, such activation of PPARα reflects a compensatory overexpression aimed at restoring normal fatty acid-focused energy homeostasis in cardiomyocytes.

In addition to suppressed mitochondrial oxidation to acetyl-CoA, increased myocardial FFA and glucose uptake could theoretically contribute to increased glycogen and lipid accumulation in the cytosol. In human septic shock patients, 6 h after onset of shock, influx of FA and glucose was found to be suppressed, but it is unknown whether uptake is still diminished in the prolonged phase of sepsis [6]. Our study indeed showed increased expression of fatty acid transporters and GLUT1 in prolonged sepsis. However, the fatty acids myristic acid, palmitate acid and stearic acid were unchanged and hexose levels were even decreased in prolonged sepsis. In a CLP model, 48 h after sepsis induction, myocardial GLUT1 but not GLUT4 expression was upregulated, and intracellular glucose concentrations were increased [16]. An important contributing factor in glucose and fatty acid availability and uptake is the occurrence of insulin resistance which commonly develops during sepsis [54]. Insulin resistance likely reduces influx and may also partially explain the observed decrease in glucose and fatty acid oxidation [15, 55].

Once glucose and fatty acids are available in the cytosol, they must be activated for downstream mitochondrial oxidation or for storage in the cytosol. For glucose, the concentration of intermediate metabolites of glycolysis, GADP and DHA, was increased in prolonged sepsis. Conversely, expression of PFK1, a key enzyme in glycolysis, was downregulated, and pyruvate and lactate levels were unaltered, arguing against upregulated glycolysis in prolonged sepsis. This contrasts with other findings in the very acute phase of sepsis, where glycolysis in cardiomyocytes was found to be stimulated in mice 8 h after CLP [56]. Remarkably, administration of 2-deoxy-D-glucose, a glycolysis inhibitor, improved cardiac function and survival [56], again supporting a mechanistic link between sepsis-induced metabolic changes and SICM.

Myocardial ketone body concentrations were normal in prolonged sepsis, but ketolytic enzymes were suppressed. Together with the observed unaltered myocardial fatty acid and acetyl-CoA levels in prolonged sepsis, this suggests that ketone bodies do not play an important role as energy substrates in this late phase of the illness. A recent study demonstrated that pretreatment of LPS-treated mice with ketones decreased myocardial oxidative stress, enhanced mitochondrial respiratory function, and improved cardiac function [57]. Additionally, randomized controlled trials in the context of cardiogenic shock and heart-failure clearly demonstrated improvement of cardiac function and cardiac output in patients receiving ketone esters [58, 59]. As such, a downregulation of ketolysis suggests a potential causal role in the pathogenesis of reduced myocardial function during stress conditions which may open new therapeutic perspectives for SCIM.

Amino acid metabolism in prolonged sepsis presented a mixed picture, with concentrations of individual amino acids being unchanged, decreased, or increased. Metabolic changes during sepsis indeed include altered concentrations of amino acids in the blood [60], which may partially explain the varying cardiac uptake. This can potentially be attributed to the balance between their use as energy substrates, as sepsis activates proteolysis [54], their production in the context of specific cellular functions, such as neurotransmitter synthesis, or their conversion to endogenous catecholamines (e.g., tyrosine). The potential contribution of changes in amino acid concentrations to the development of SICM has been sparsely studied in recent literature. However, myocardial function did improve in a CLP rat model of sepsis through the administration of glutamine, again suggesting a potential causal link between SICM and metabolic substrate utilization for energy production [61].

Our study has several limitations. First, it lacks data on myocardial function, protein expression and enzymatic activity, and enzymatic activity, as well as direct measurements of mitochondrial function. Secondly, although we observed changes in gene expression of metabolic enzymes, caution is warranted when interpreting these findings as direct indicators of enzymatic activity, as gene expression does not necessarily correlate with functional protein changes or activity levels. Lastly, we have used a mouse model to investigate the complex context of human sepsis. In addition, we cannot exclude selection bias as animals that did not survive the pre-aimed study period of 5 days were not included in the analyses. However, many of our observations in acute sepsis, indicating suppressed mitochondrial oxidation and accumulation of glycogen and lipids in cardiomyocytes, align with human literature. On the other hand, gene expression of specific metabolic enzymes often differed from those observed in other models. It should be noted that existing models are highly heterogeneous, varying in the species used (mice vs. rats), the type of sepsis induction (LPS vs. CLP), the time frame in the disease course after sepsis induction, and whether and how the models were resuscitated and fed. Each model has unique characteristics with specific attempts to mimic human sepsis, but each also has its limitations [8]. A CLP mouse model of sepsis, however, more closely approximates human sepsis than a more transient hit by LPS injections [18]. In addition, we provided intravenous resuscitation and treatment with antibiotics, analgesics and nutrition to the septic mice, which allowed to study also the prolonged phase of illness in a clinically relevant model that mimics the typical metabolic and inflammatory changes observed in human prolonged septic patients [62, 63].

In conclusion, this study demonstrated that most metabolic changes observed in acute sepsis persist in prolonged sepsis. In the acute phase of sepsis, these changes, suggesting suppressed mitochondrial oxidation and accumulation of lipids and glycogen in the cytosol, have been interpreted as potentially adaptive, preventing acute cell death in patients with SICM. However, the observation of a persistent inflammatory response and sustained metabolic dysregulation in prolonged sepsis may contribute to later sequelae in patients with SICM, which could open perspectives for prevention or treatment. Future studies should investigate the functional impact of these sepsis-induced myocardial alterations in prolonged sepsis as well as its causal role in potential long-term sequelae of SICM.

## Supplementary Information

Additional file 1

## Data Availability

The datasets used and/or analyzed during the current study are available from the corresponding author on reasonable request.

## Abbreviations

CLP: Cecal ligation and puncture
LPS: Lipopolysaccharide
SICM: Sepsis-induced cardiomyopathy

## References

[1] L’Heureux M, Sternberg M, Brath L, Turlington J, Kashiouris MG (2020) Sepsis-induced cardiomyopathy: a comprehensive review. Curr Cardiol Rep 22(5):35 32377972 10.1007/s11886-020-01277-2 PMC7222131

[2] Hasegawa D, Ishisaka Y, Maeda T, Prasitlumkum N, Nishida K, Dugar S, Sato R (2023) Prevalence and prognosis of sepsis-induced cardiomyopathy: a systematic review and meta-analysis. J Intensive Care Med 38(9):797–808 37272081 10.1177/08850666231180526

[3] Vincent JL, Gris P, Coffernils M, Leon M, Pinsky M, Reuse C, Kahn RJ (1992) Myocardial depression characterizes the fatal course of septic shock. Surgery 111(6):660–667 1595062

[4] Landesberg G, Gilon D, Meroz Y, Georgieva M, Levin PD, Goodman S, Avidan A, Beeri R, Weissman C, Jaffe AS et al (2012) Diastolic dysfunction and mortality in severe sepsis and septic shock. Eur Heart J 33(7):895–903 21911341 10.1093/eurheartj/ehr351 PMC3345552

[5] Martin L, Derwall M, Al Zoubi S, Zechendorf E, Reuter DA, Thiemermann C, Schuerholz T (2019) The septic heart: current understanding of molecular mechanisms and clinical implications. Chest 155(2):427–437 30171861 10.1016/j.chest.2018.08.1037

[6] Dhainaut JF, Huyghebaert MF, Monsallier JF, Lefevre G, Dall’Ava-Santucci J, Brunet F, Villemant D, Carli A, Raichvarg D (1987) Coronary hemodynamics and myocardial metabolism of lactate, free fatty acids, glucose, and ketones in patients with septic shock. Circulation 75(3):533–541 3815765 10.1161/01.cir.75.3.533

[7] Hollenberg SM, Singer M (2021) Pathophysiology of sepsis-induced cardiomyopathy. Nat Rev Cardiol 18(6):424–434 33473203 10.1038/s41569-020-00492-2

[8] Stanzani G, Duchen MR, Singer M (2019) The role of mitochondria in sepsis-induced cardiomyopathy. Biochim Biophys Acta Mol Basis Dis 1865(4):759–773 30342158 10.1016/j.bbadis.2018.10.011

[9] Pruszczyk A, Zawadka M, Andruszkiewicz P, La Via L, Herpain A, Sato R, Dugar S, Chew MS, Sanfilippo F (2023) Mortality in patients with septic cardiomyopathy identified by longitudinal strain by speckle tracking echocardiography: an updated systematic review and meta-analysis with trial sequential analysis. Anaesth Crit Care Pain Med 43:101339 38128732 10.1016/j.accpm.2023.101339

[10] Smeding L, Plotz FB, Groeneveld AB, Kneyber MC (2012) Structural changes of the heart during severe sepsis or septic shock. Shock 37(5):449–456 22301606 10.1097/SHK.0b013e31824c3238

[11] Takasu O, Gaut JP, Watanabe E, To K, Fagley RE, Sato B, Jarman S, Efimov IR, Janks DL, Srivastava A et al (2013) Mechanisms of cardiac and renal dysfunction in patients dying of sepsis. Am J Respir Crit Care Med 187(5):509–517 23348975 10.1164/rccm.201211-1983OC PMC3733408

[12] Rossi MA, Celes MR, Prado CM, Saggioro FP (2007) Myocardial structural changes in long-term human severe sepsis/septic shock may be responsible for cardiac dysfunction. Shock 27(1):10–18 17172974 10.1097/01.shk.0000235141.05528.47

[13] Rastaldo R, Pagliaro P, Cappello S, Penna C, Mancardi D, Westerhof N, Losano G (2007) Nitric oxide and cardiac function. Life Sci 81(10):779–793 17707439 10.1016/j.lfs.2007.07.019

[14] Fliers E, Bianco AC, Langouche L, Boelen A (2015) Thyroid function in critically ill patients. Lancet Diabetes Endocrinol 3(10):816–825 26071885 10.1016/S2213-8587(15)00225-9 PMC4979220

[15] Wasyluk W, Nowicka-Stazka P, Zwolak A (2021) Heart metabolism in sepsis-induced cardiomyopathy-unusual metabolic dysfunction of the heart. Int J Environ Res Public Health 18(14):7598 34300048 10.3390/ijerph18147598 PMC8303349

[16] Levy RJ, Piel DA, Acton PD, Zhou R, Ferrari VA, Karp JS, Deutschman CS (2005) Evidence of myocardial hibernation in the septic heart. Crit Care Med 33(12):2752–2756 16352955 10.1097/01.ccm.0000189943.60945.77

[17] Schilling J, Lai L, Sambandam N, Dey CE, Leone TC, Kelly DP (2011) Toll-like receptor-mediated inflammatory signaling reprograms cardiac energy metabolism by repressing peroxisome proliferator-activated receptor gamma coactivator-1 signaling. Circ Heart Fail 4(4):474–482 21558447 10.1161/CIRCHEARTFAILURE.110.959833 PMC3144030

[18] Standage SW, Bennion BG, Knowles TO, Ledee DR, Portman MA, McGuire JK, Liles WC, Olson AK (2017) PPARalpha augments heart function and cardiac fatty acid oxidation in early experimental polymicrobial sepsis. Am J Physiol Heart Circ Physiol 312(2):H239–H249 27881386 10.1152/ajpheart.00457.2016 PMC6734063

[19] Parker MM, Shelhamer JH, Bacharach SL, Green MV, Natanson C, Frederick TM, Damske BA, Parrillo JE (1984) Profound but reversible myocardial depression in patients with septic shock. Ann Intern Med 100(4):483–490 6703540 10.7326/0003-4819-100-4-483

[20] Jardin F, Fourme T, Page B, Loubieres Y, Vieillard-Baron A, Beauchet A, Bourdarias JP (1999) Persistent preload defect in severe sepsis despite fluid loading: a longitudinal echocardiographic study in patients with septic shock. Chest 116(5):1354–1359 10559099 10.1378/chest.116.5.1354

[21] Zhang L, Qi D, Peng M, Meng B, Wang X, Zhang X, Zuo Z, Li L, Wang Z, Zou W et al (2023) Decoding molecular signature on heart of septic mice with distinct left ventricular ejection fraction. iScience 26(10):107825 37736036 10.1016/j.isci.2023.107825 PMC10509301

[22] Lima MR, Silva D (2023) Septic cardiomyopathy: a narrative review. Rev Port Cardiol 42(5):471–481 36893835 10.1016/j.repc.2021.05.020

[23] Carbone F, Liberale L, Preda A, Schindler TH, Montecucco F (2022) Septic cardiomyopathy: from pathophysiology to the clinical setting. Cells 11(18):2833 36139408 10.3390/cells11182833 PMC9496713

[24] Langouche L, Teblick A, Gunst J, Van den Berghe G (2023) The hypothalamus-pituitary-adrenocortical response to critical illness: a concept in need of revision. Endocr Rev 44:1096–1106 37409973 10.1210/endrev/bnad021 PMC10638597

[25] Preiser JC, Ichai C, Orban JC, Groeneveld AB (2014) Metabolic response to the stress of critical illness. Br J Anaesth 113(6):945–954 24970271 10.1093/bja/aeu187

[26] Vallabhajosyula S, Shankar A, Vojjini R, Cheungpasitporn W, Sundaragiri PR, DuBrock HM, Sekiguchi H, Frantz RP, Cajigas HR, Kane GC et al (2021) Impact of right ventricular dysfunction on short-term and long-term mortality in sepsis: a meta-analysis of 1,373 patients. Chest 159(6):2254–2263 33359215 10.1016/j.chest.2020.12.016 PMC8579312

[27] Beesley SJ, Sorensen J, Walkey AJ, Tonna JE, Lanspa MJ, Hirshberg E, Grissom CK, Horne BD, Burk R, Abraham TP et al (2021) Long-term implications of abnormal left ventricular strain during sepsis. Crit Care Med 49(4):e444–e453 33591007 10.1097/CCM.0000000000004886 PMC7996634

[28] Kilkenny C, Browne WJ, Cuthill IC, Emerson M, Altman DG (2010) Improving bioscience research reporting: the ARRIVE guidelines for reporting animal research. PLoS Biol 8(6):e1000412 20613859 10.1371/journal.pbio.1000412 PMC2893951

[29] Derde S, Thiessen S, Goossens C, Dufour T, Van den Berghe G, Langouche L (2017) Use of a central venous line for fluids, drugs and nutrient administration in a mouse model of critical illness. J Vis Exp (123) 10.3791/55553 PMC5565154 28518095

[30] Hoffman M, Kyriazis ID, Lucchese AM, de Lucia C, Piedepalumbo M, Bauer M, Schulze PC, Bonios MJ, Koch WJ, Drosatos K (2019) Myocardial strain and cardiac output are preferable measurements for cardiac dysfunction and can predict mortality in septic mice. J Am Heart Assoc 8(10):e012260 31112430 10.1161/JAHA.119.012260 PMC6585345

[31] Hoyer FF, Naxerova K, Schloss MJ, Hulsmans M, Nair AV, Dutta P, Calcagno DM, Herisson F, Anzai A, Sun Y et al (2019) Tissue-specific macrophage responses to remote injury impact the outcome of subsequent local immune challenge. Immunity 51(5):899–914.e897 10.1016/j.immuni.2019.10.010 PMC6892583 31732166

[32] Zeng XM, Liu DH, Han Y, Huang ZQ, Zhang JW, Huang Q (2020) Assessment of inflammatory markers and mitochondrial factors in a rat model of sepsis-induced myocardial dysfunction. Am J Transl Res 12(3):901–911 32269722 PMC7137057

[33] Kumar A, Thota V, Dee L, Olson J, Uretz E, Parrillo JE (1996) Tumor necrosis factor alpha and interleukin 1beta are responsible for in vitro myocardial cell depression induced by human septic shock serum. J Exp Med 183(3):949–958 8642298 10.1084/jem.183.3.949 PMC2192364

[34] Weisensee D, Bereiter-Hahn J, Schoeppe W, Low-Friedrich I (1993) Effects of cytokines on the contractility of cultured cardiac myocytes. Int J Immunopharmacol 15(5):581–587 8375940 10.1016/0192-0561(93)90075-a

[35] Hosenpud JD, Campbell SM, Mendelson DJ (1989) Interleukin-1-induced myocardial depression in an isolated beating heart preparation. J Heart Transplant 8(6):460–464 2614547

[36] Busch K, Kny M, Huang N, Klassert TE, Stock M, Hahn A, Graeger S, Todiras M, Schmidt S, Chamling B et al (2021) Inhibition of the NLRP3/IL-1beta axis protects against sepsis-induced cardiomyopathy. J Cachexia Sarcopenia Muscle 12(6):1653–1668 34472725 10.1002/jcsm.12763 PMC8718055

[37] Fujimura K, Karasawa T, Komada T, Yamada N, Mizushina Y, Baatarjav C, Matsumura T, Otsu K, Takeda N, Mizukami H et al (2023) NLRP3 inflammasome-driven IL-1beta and IL-18 contribute to lipopolysaccharide-induced septic cardiomyopathy. J Mol Cell Cardiol 180:58–68 37172930 10.1016/j.yjmcc.2023.05.003

[38] Zhang H, Wang HY, Bassel-Duby R, Maass DL, Johnston WE, Horton JW, Tao W (2007) Role of interleukin-6 in cardiac inflammation and dysfunction after burn complicated by sepsis. Am J Physiol Heart Circ Physiol 292(5):H2408–H2416 17220181 10.1152/ajpheart.01150.2006

[39] Pathan N, Hemingway CA, Alizadeh AA, Stephens AC, Boldrick JC, Oragui EE, McCabe C, Welch SB, Whitney A, O’Gara P et al (2004) Role of interleukin 6 in myocardial dysfunction of meningococcal septic shock. Lancet 363(9404):203–209 14738793 10.1016/S0140-6736(03)15326-3

[40] Zhang K, Wang Y, Chen S, Mao J, Jin Y, Ye H, Zhang Y, Liu X, Gong C, Cheng X et al (2023) TREM2(hi) resident macrophages protect the septic heart by maintaining cardiomyocyte homeostasis. Nat Metab 5(1):129–146 36635449 10.1038/s42255-022-00715-5 PMC9886554

[41] Peng Y, Yang Q, Gao S, Liu Z, Kong W, Bian X, Li Z, Ye J (2022) IL-6 protects cardiomyocytes from oxidative stress at the early stage of LPS-induced sepsis. Biochem Biophys Res Commun 603:144–152 35290918 10.1016/j.bbrc.2022.03.013

[42] Drosatos K, Khan RS, Trent CM, Jiang H, Son NH, Blaner WS, Homma S, Schulze PC, Goldberg IJ (2013) Peroxisome proliferator-activated receptor-gamma activation prevents sepsis-related cardiac dysfunction and mortality in mice. Circ Heart Fail 6(3):550–562 23572494 10.1161/CIRCHEARTFAILURE.112.000177 PMC3690188

[43] Drosatos K, Drosatos-Tampakaki Z, Khan R, Homma S, Schulze PC, Zannis VI, Goldberg IJ (2011) Inhibition of c-Jun-N-terminal kinase increases cardiac peroxisome proliferator-activated receptor alpha expression and fatty acid oxidation and prevents lipopolysaccharide-induced heart dysfunction. J Biol Chem 286(42):36331–36339 21873422 10.1074/jbc.M111.272146 PMC3196095

[44] Xia C, Dong R, Chen C, Wang H, Wang DW (2015) Cardiomyocyte specific expression of Acyl-coA thioesterase 1 attenuates sepsis induced cardiac dysfunction and mortality. Biochem Biophys Res Commun 468(4):533–540 26518651 10.1016/j.bbrc.2015.10.078

[45] Derde S, Vanhorebeek I, Guiza F, Derese I, Gunst J, Fahrenkrog B, Martinet W, Vervenne H, Ververs EJ, Larsson L et al (2012) Early parenteral nutrition evokes a phenotype of autophagy deficiency in liver and skeletal muscle of critically ill rabbits. Endocrinology 153(5):2267–2276 22396453 10.1210/en.2011-2068

[46] Hotchkiss RS, Song SK, Neil JJ, Chen RD, Manchester JK, Karl IE, Lowry OH, Ackerman JJ (1991) Sepsis does not impair tricarboxylic acid cycle in the heart. Am J Physiol 260(1 Pt 1):C50-57 1987781 10.1152/ajpcell.1991.260.1.C50

[47] McDonough KH, Henry JJ, Lang CH, Spitzer JJ (1986) Substrate utilization and high energy phosphate levels of hearts from hyperdynamic septic rats. Circ Shock 18(3):161–170 3698211

[48] Watts JA, Kline JA, Thornton LR, Grattan RM, Brar SS (2004) Metabolic dysfunction and depletion of mitochondria in hearts of septic rats. J Mol Cell Cardiol 36(1):141–150 14734056 10.1016/j.yjmcc.2003.10.015

[49] Supinski GS, Callahan LA (2006) Polyethylene glycol-superoxide dismutase prevents endotoxin-induced cardiac dysfunction. Am J Respir Crit Care Med 173(11):1240–1247 16514113 10.1164/rccm.200410-1346OC PMC2662969

[50] Supinski GS, Murphy MP, Callahan LA (2009) MitoQ administration prevents endotoxin-induced cardiac dysfunction. Am J Physiol Regul Integr Comp Physiol 297(4):R1095-1102 19657095 10.1152/ajpregu.90902.2008 PMC2763820

[51] Singer M (2017) Critical illness and flat batteries. Crit Care 21(Suppl 3):309 29297363 10.1186/s13054-017-1913-9 PMC5751585

[52] Feingold K, Kim MS, Shigenaga J, Moser A, Grunfeld C (2004) Altered expression of nuclear hormone receptors and coactivators in mouse heart during the acute-phase response. Am J Physiol Endocrinol Metab 286(2):E201-207 14701665 10.1152/ajpendo.00205.2003

[53] Zhu XX, Wang X, Jiao SY, Liu Y, Shi L, Xu Q, Wang JJ, Chen YE, Zhang Q, Song YT et al (2023) Cardiomyocyte peroxisome proliferator-activated receptor alpha prevents septic cardiomyopathy via improving mitochondrial function. Acta Pharmacol Sin 44(11):2184–2200 37328648 10.1038/s41401-023-01107-5 PMC10618178

[54] Song J, Fang X, Zhou K, Bao H, Li L (2023) Sepsis‑induced cardiac dysfunction and pathogenetic mechanisms. Mol Med Rep 28(6) 10.3892/mmr.2023.13114 PMC10619129 37859613

[55] Drosatos K, Lymperopoulos A, Kennel PJ, Pollak N, Schulze PC, Goldberg IJ (2015) Pathophysiology of sepsis-related cardiac dysfunction: driven by inflammation, energy mismanagement, or both? Curr Heart Fail Rep 12(2):130–140 25475180 10.1007/s11897-014-0247-z PMC4474734

[56] Zheng Z, Ma H, Zhang X, Tu F, Wang X, Ha T, Fan M, Liu L, Xu J, Yu K et al (2017) Enhanced glycolytic metabolism contributes to cardiac dysfunction in polymicrobial sepsis. J Infect Dis 215(9):1396–1406 28368517 10.1093/infdis/jix138 PMC5451607

[57] Ji L, He Q, Liu Y, Deng Y, Xie M, Luo K, Cai X, Zuo Y, Wu W, Li Q et al (2022) Ketone body beta-hydroxybutyrate prevents myocardial oxidative stress in septic cardiomyopathy. Oxid Med Cell Longev 2022:2513837 35340211 10.1155/2022/2513837 PMC8956399

[58] Berg-Hansen K, Christensen KH, Gopalasingam N, Nielsen R, Eiskjaer H, Moller N, Birkelund T, Christensen S, Wiggers H (2023) Beneficial effects of ketone ester in patients with cardiogenic shock: a randomized, controlled, double-blind trial. JACC Heart Fail 11(10):1337–1347 37452805 10.1016/j.jchf.2023.05.029

[59] Berg-Hansen K, Gopalasingam N, Christensen KH, Ladefoged B, Andersen MJ, Poulsen SH, Borlaug BA, Nielsen R, Moller N, Wiggers H (2024) Cardiovascular effects of oral ketone ester treatment in patients with heart failure with reduced ejection fraction: a randomized, controlled, double-blind trial. Circulation 149(19):1474–1489 38533643 10.1161/CIRCULATIONAHA.123.067971 PMC11081479

[60] Su L, Li H, Xie A, Liu D, Rao W, Lan L, Li X, Li F, Xiao K, Wang H et al (2015) Dynamic changes in amino acid concentration profiles in patients with sepsis. PLoS ONE 10(4):e0121933 25849571 10.1371/journal.pone.0121933 PMC4388841

[61] Groening P, Huang Z, La Gamma EF, Levy RJ (2011) Glutamine restores myocardial cytochrome C oxidase activity and improves cardiac function during experimental sepsis. JPEN J Parenter Enteral Nutr 35(2):249–254 21378254 10.1177/0148607110383040

[62] Thiessen SE, Derde S, Derese I, Dufour T, Vega CA, Langouche L, Goossens C, Peersman N, Vermeersch P, Vander Perre S et al (2017) Role of glucagon in catabolism and muscle wasting of critical illness and modulation by nutrition. Am J Respir Crit Care Med 196(9):1131–1143 28475354 10.1164/rccm.201702-0354OC

[63] Goossens C, Weckx R, Derde S, Dufour T, Vander Perre S, Pauwels L, Thiessen SE, Van Veldhoven PP, Van den Berghe G, Langouche L (2019) Adipose tissue protects against sepsis-induced muscle weakness in mice: from lipolysis to ketones. Crit Care 23(1):236 31262340 10.1186/s13054-019-2506-6 PMC6600878

