# Supplementary material for: A murine model of acute and prolonged abdominal sepsis, supported by intensive care, reveals time-dependent metabolic alterations in the heart
Source: Intensive Care Med Exp. 2025 Jan 17;13:6. doi: 10.1186/s40635-025-00715-1 (PMC11748666; doi:10.1186/s40635-025-00715-1)
Supplement: Supplementary file 1 — Additional file 1 [file 40635_2025_715_MOESM1_ESM.docx]

**Supplementary Materials**

**Table S1 - Gene expression kits**

| Symbol | Gene Name | Number Gene expression assay Thermo Fisher Scientific |
| --- | --- | --- |
|  | Figure 1: Inflammatory markers |  |
| Tnf | tumor necrosis factor | Mm00443258_m1 |
| Il1b | interleukin 1 beta | Mm00434228_m1 |
| Il6 | Interleukin 6 | Mm00446190_m1 |
|  | Figure 2: Fatty acid metabolism |  |
| FAT/Cd36 | Fatty acid translocase/CD36 antigen | Mm00432403_m1 |
| Slc27a1 | Solute carrier family 27 (fatty acid transporter), member 1 (FATP1) | Mm00449511_m1 |
| Acot1 | Acyl-CoA thioesterase 1 | Mm01622471_s1 |
| Ppara | Peroxisome proliferator activated receptor alpha | Mm00440936_m1 |
| Ppargc1a | Peroxisome proliferative activated receptor, gamma, coactivator 1 alpha | Mm01208835_m1 |
| Cpt1b | Carnitine palmitoyltransferase 1b | Mm00487191_g1 |
| Acadm | Acyl-Coenzyme A dehydrogenase, medium chain | Mm01323360_g1 |
| Acadl | Acyl-Coenzyme A dehydrogenase, long chain | Mm00599660_m1 |
| Acaca | Acetyl-CoA carboxylase alpha (ACC1) | Mm01304258_m1 |
| Mlycd | malonyl-CoA decarboxylase (MCD) | Mm01245665_m1 |
| Dgat1 | diacylglycerol O-acyltransferase 1 | Mm00515643_m1 |
| Pnlpa2 | patatin-like phospholipase domain containing 2 (ATGL) | Mm00503040_m1 |
| Lipe | lipase, hormone sensitive | Mm00495359_m1 |
|  | Figure 3: Glucose metabolism |  |
| Slc2a1 | solute carrier family 2 (facilitated glucose transporter), member 1 (GLUT1) | Mm00441473_m1 |
| Slc2a4 | solute carrier family 2 (facilitated glucose transporter), member 4 (GLUT4) | Mm00436615_m1 |
| Gys1 | glycogen synthase 1, muscle | Mm01962575_s1 |
| Gsk3a | glycogen synthase kinase 3 alpha | Mm01719731_g1 |
| Pygb | brain glycogen phosphorylase | Mm00464080_m1 |
| Pfkm | Phosphofructokinase, muscle | Mm01309576_m1 |
| Pfkfb2 | 6-phosphofructo-2-kinase/fructose-2,6-biphosphatase 2 (PDP2) | Mm00435575_m1 |
| Pfkfb3 | 6-phosphofructo-2-kinase/fructose-2,6-biphosphatase 3 (PDP3) | Mm00504650_m1 |
| Ldhb | lactate dehydrogenase B | Mm05874166_g1 |
| Mpc1 | mitochondrial pyruvate carrier 1 | Mm00834592_g1 |
| Mcp2 | mitochondrial pyruvate carrier 2 | Mm00770996_g1 |
| Pdha1 | pyruvate dehydrogenase E1 alpha 1 | Mm00468678_m1 |
| Pdhb | pyruvate dehydrogenase (lipoamide) E1 beta | Mm00499323_m1 |
| Pdk1 | pyruvate dehydrogenase kinase, isoenzyme 1 | Mm00554300_m1 |
| Pdk3 | pyruvate dehydrogenase kinase, isoenzyme 3 | Mm00455220_m1 |
|  | Figure 4: Ketone metabolism |  |
| Bdh1 | 3-hydroxybutyrate dehydrogenase, type 1 | Mm00558330_m1 |
| Oxct1 | 3-oxoacid CoA transferase 1 (SCOT) | Mm00499303_m1 |
| Acat1 | acetyl-Coenzyme A acetyltransferase 1 | Mm00507463_m1 |
| Hmgcs2 | 3-hydroxy-3-methylglutaryl-Coenzyme A synthase 2 | Mm00550050_m1 |
|  | Figure 5: amino acid metabolism |  |
| Bckdha | branched chain ketoacid dehydrogenase E1, alpha polypeptide | Mm00476112_m1 |
| Bckdhb | branched chain ketoacid dehydrogenase E1, beta polypeptide | Mm01177077_m1 |
| Bckdk | branched chain ketoacid dehydrogenase kinase | Mm00437777_m1 |
